# HIF-1α Activation by Intermittent Hypoxia Requires NADPH Oxidase Stimulation by Xanthine Oxidase

**DOI:** 10.1371/journal.pone.0119762

**Published:** 2015-03-09

**Authors:** Jayasri Nanduri, Damodara Reddy Vaddi, Shakil A. Khan, Ning Wang, Vladislav Makarenko, Gregg L. Semenza, Nanduri R. Prabhakar

**Affiliations:** 1 Institute for Integrative Physiology and Center for Systems Biology of O2 Sensing, Biological Sciences Division, University of Chicago, Chicago, Illinois, United States of America; 2 Vascular Program, Institute for Cell Engineering; Department of Pediatrics, Medicine, Oncology, Radiation Oncology and Biological Chemistry; and Mckusick-Nathans Institute of Genetic Medicine, The Johns Hopkins University School of Medicine, Baltimore, Maryland, United States of America; Goethe Universität Frankfurt, GERMANY

## Abstract

Hypoxia-inducible factor 1 (HIF-1) mediates many of the systemic and cellular responses to intermittent hypoxia (IH), which is an experimental model that simulates O_2_ saturation profiles occurring with recurrent apnea. IH-evoked HIF-1α synthesis and stability are due to increased reactive oxygen species (ROS) generated by NADPH oxidases, especially Nox2. However, the mechanisms by which IH activates Nox2 are not known. We recently reported that IH activates xanthine oxidase (XO) and the resulting increase in ROS elevates intracellular calcium levels. Since Nox2 activation requires increased intracellular calcium levels, we hypothesized XO-mediated calcium signaling contributes to Nox activation by IH. We tested this possibility in rat pheochromocytoma PC12 cells subjected to IH consisting alternating cycles of hypoxia (1.5% O_2_ for 30 sec) and normoxia (21% O_2_ for 5 min). Kinetic analysis revealed that IH-induced XO preceded Nox activation. Inhibition of XO activity either by allopurinol or by siRNA prevented IH-induced Nox activation, translocation of the cytosolic subunits p47^phox^ and p67^phox^ to the plasma membrane and their interaction with gp91^phox^. ROS generated by XO also contribute to IH-evoked Nox activation via calcium-dependent protein kinase C stimulation. More importantly, silencing XO blocked IH-induced upregulation of HIF-1α demonstrating that HIF-1α activation by IH requires Nox2 activation by XO.

## Introduction

Sleep disordered breathing with recurrent apnea is characterized by transient (~10–15 sec in adults) repetitive cessations of breathing, resulting in periodic decreases in arterial blood O_2_ or intermittent hypoxia (IH). Patients with recurrent apnea develop several co-morbidities including hypertension and breathing abnormalities [1, 2]. Hypoxia-inducible factor-1 (HIF-1) is the master transcriptional activator that regulates gene expression during hypoxia [3]. IH activates HIF-1 mediated transcription in cell cultures and rodents [4–6]. Mice with heterozygous deficiency of HIF-1α exhibit remarkable absence of IH-induced hypertension, and breathing abnormalities [5, 6], suggesting that activation of HIF-1 contributes to the cardio-respiratory abnormalities caused by IH. Analysis of the mechanisms underlying HIF-1 activation showed that IH activates NADPH oxidase (Nox), especially Nox2 [7]. Disruption of Nox2 function, prevents HIF-1 activation by IH in cell cultures and in mice [4, 8], suggesting that Nox2 is critical for HIF-1 activation by IH. However, the mechanism by which IH activates Nox2 has not been examined.

Nox2 is a multi-enzyme protein complex consisting of a membrane-bound gp91^phox^ and cytosolic p67^phox^ and p47^phox^ subunits [9–11]. Nox2 activation requires phosphorylation of cytosolic subunits by Ca^2+^-activated protein kinase C (PKC) and translocation to the membrane to form a complex with gp91^phox^ [12]. We recently reported that IH activates xanthine oxidase (XO), which is a major cellular source of reactive oxygen species (ROS) [13]. IH-induced XO activation leads to ROS-dependent elevation of intracellular calcium ([Ca^2+^]_i_). Given that Nox2 activation critically depends on Ca^2+^-activated PKC [14], we hypothesized that [Ca^2+^]_i_ elevation by XO mediates IH-induced Nox2 activation and subsequent stimulation of HIF-1. We examined this possibility in rat pheochromocytoma 12 (PC12) cell cultures exposed to IH.

## Materials and Methods

### Exposure of cell cultures to IH

PC12 cells (original clone from Dr. Lloyd Greene, Columbia University Medical Center, New York) [15] were cultured in Dulbecco’s modified Eagle’s medium (DMEM) supplemented with 10% horse serum, 5% fetal bovine serum (FBS), penicillin (100 U/ml), and streptomycin (100 μg/ml) under 10% CO_2_ and 90% air (20% O_2_) at 37°C [16]. Experiments were performed on cells serum starved for 16 h in antibiotic free medium. In the experiments involving treatment with drugs, cells were pre-incubated for 30 min with either drug or vehicle. Cell cultures were exposed to IH (1.5% O_2_ for 30 sec followed by 20% O_2_ for 5 min at 37°C) as described [16]. Ambient O_2_ levels in the IH chamber were monitored by an O_2_ analyzer (Alpha Omega Instruments).

### Studies with short interfering RNA (siRNA)

PC12 cells (5x10^5^) were plated on culture dishes coated with type IV collagen (BD Biosciences, Bedford, MA) and transfected with siRNA (Santa Cruz) targeted to XDH, gp91^phox^ (Nox2), or a scrambled sequence at a concentration of 100 pmol/mL using DharmaFECT 2 (Dharmacon Research). Transfected cells were cultured in complete medium for 48 h before being exposed to IH.

### Measurement of XO activity

Amplex Red Xanthine/Xanthine oxidase assay kit (Molecular Probes) was used to monitor to XO activity as described [13]. Cell lysates were incubated with a reaction mixture containing hypoxanthine, horseradish peroxidase (HRP) and Amplex Red. H_2_O_2_ reacts with Amplex Red in the presence of HRP to generate the red fluorescent oxidation product resorufin. Fluorescence was measured by excitation at 530 nm and emission at 590 nm. The concentration of XO in samples was determined from a standard curve and expressed as XO mU/mg protein.

### Measurement of NADPH oxidase activity

NADPH oxidase activity in the membrane-enriched protein fractions was measured by superoxide dismutase-inhibitable rate of cytochrome c reduction as described [17]. Briefly, the assay medium contained 100 μg membrane protein, 150 μm cytochrome c and 100 μm NADPH in 25 mM HEPES buffer (pH 7.0). The assay was performed in the presence and absence of superoxide dismutase (200 units/ml) at 37°C for 30 min. Cytochrome c reduction was measured by reading the absorbance at 550 nm. NADPH oxidase activity was calculated based on extinction coefficient [21mmol/(L)] per cm and expressed as nmol/min/mg protein.

### Immunoprecipitation and Immunoblot assays

Membrane enriched fractions from PC12 cells were isolated using a plasma membrane protein extraction kit (Abcam). Briefly, cells were homogenized in the buffer mix provided and centrifuged at 700 *g* for 10 min at 4°C. The supernatant was further centrifuged at 10,000 *g* for 30 minutes at 4°C. The pellet contained the plasma membrane and cellular organelle membrane proteins. The purified membrane enriched proteins were resuspended in lysis buffer and fractionated by polyacrylamide-SDS gel electrophoresis and immunoblotted with gp91^phox^ (sc-7663), p67^phox^ (sc-7660) or p47^phox^ (sc-3678) antibodies (Santa Cruz Biotechnology Inc, Dallas, USA). Antibody binding was detected using HRP-conjugated secondary antibodies followed by enhanced chemiluminescence detection system (Bio-Rad). Immunoprecipitation experiments were performed using protein A/G magnetic beads (Millipore). To detect phosphorylated p47^phox^ and p67^phox^, total cell lysates were incubated with p47^phox^ and p67^phox^ antibodies and immune complexes were isolated using protein A/G magnetic beads. Phosphorylation of p47^phox^ and p67^phox^ was analyzed by immunoblots with an anti-phosphoserine antibody at a 1:500 dilution.

### Measurement of PKC activity

The PKC activity was determined using an ELISA kit (Enzo Life sciences, Farmingdale, NY, USA), according to the manufacturer's instructions. Briefly, lysates from control or IH exposed cells were added to a microplate that was precoated with a PKC substrate, followed by the addition of ATP to initiate the reaction. PKC activity was determined by adding a phospho-specific substrate antibody conjugated to peroxidase and tetramethylbenzidine as a substrate. Color development, which is proportional to PKC activity was measured at 450-nm absorbance.

### Measurement of [Ca^2+^]_i_

Intracellular calcium levels were monitored in PC12 cells using Fura-2-AM as described previously [18]. Background fluorescence was subtracted from signals. Image intensity at 340 nm was divided by 380-nm image intensity to obtain the ratiometric image. Ratios were converted to free [Ca^2+^]_i_ using calibration curves constructed *in vitro* by adding Fura-2 (50 μM, free acid) to solutions containing known concentrations of Ca^2+^ (0–2000 nM).

### Statistical analysis

Data were expressed as mean ± S.E.M from 3–5 independent experiments with each performed in triplicate. Statistical analysis was performed by analysis of variance (ANOVA) and *p* values < 0.05 were considered significant.

## Results

### XO mediates IH-induced Nox activation

**Fig 1:**
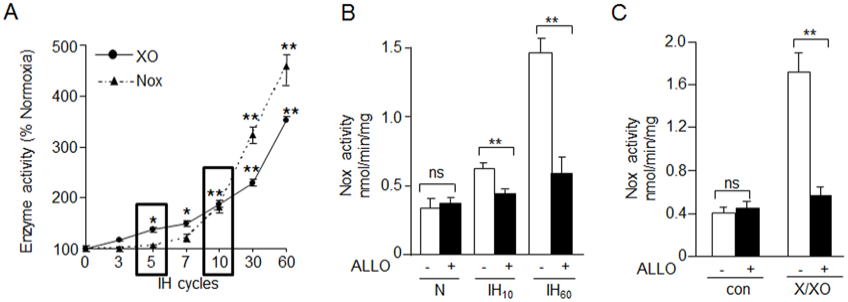
XO activation is required for increased Nox activity in response to IH. A) Time course of IH on XO and Nox activity. Squares represent significant increase in XO and Nox activity at 5 and 10 cycles of IH respectively. B) Effect of XO inhibitor, Allopurinol (ALLO; 10 μM) on Nox activity in PC12 cells exposed to 10 cycles (IH_10_) and 60 cycles (IH_60_) of IH or normoxia (N). C) Effect of Xanthine/XO (250 μM/0.01 U/mL) treatment on Nox activity under normoxic conditions with and without ALLO. Data is presented as mean ± S.E.M from four independent experiments. ** p<0.01; * p< 0.05; ns: not significant.

PC12 cells were exposed to increasing cycles of IH with each cycle consisting of 30 sec of hypoxia followed by 5 min of normoxia. Analysis of the time course revealed that XO activity increased progressively with increasing number of IH cycles with a significant activation after 5 cycles, whereas Nox activation required a minimum of 10 cycles of IH (Fig. 1A). Because XO activation by IH precedes that of Nox, we hypothesized that it triggers Nox activation. To test this possibility, PC12 cells were treated with 10 μM allopurinol (ALLO), an inhibitor of XO and then were exposed to IH. ALLO blocked Nox activation in cells exposed to either IH_10_ or IH_60_ (Fig. 1B). Conversely, treating control cells with Xanthine/XO, (250 μM/0.01 U/mL), stimulated Nox activity and ALLO blocked this effect (Fig. 1C).

**Fig 2:**
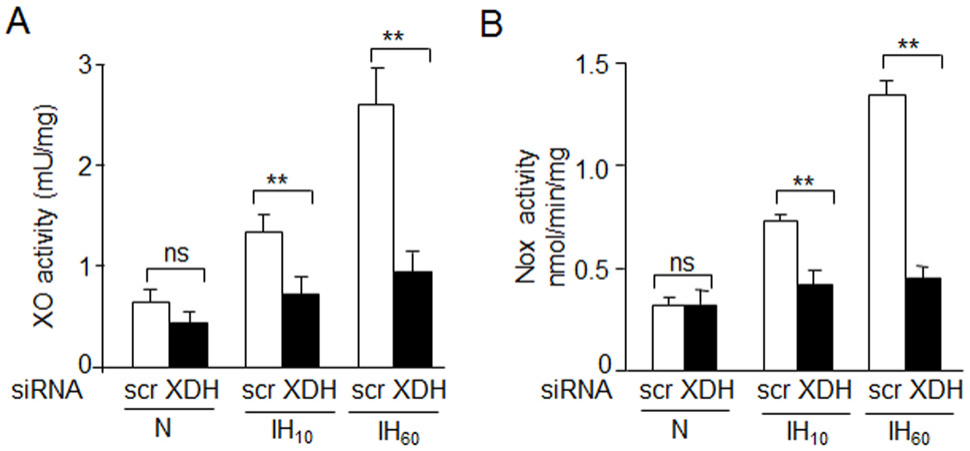
Effect of silencing XO on Nox activation. A-B) XO and Nox activity in PC12 cells transfected with siRNA targeted against XDH, the precursor of XO or scrambled (scr) siRNA and exposed to IH_10,_ IH_60_ or normoxia (N). Data is presented as mean ± S.E.M from four independent experiments. **p<0.01; ns: not significant.

To further establish the role of XO, PC12 cells were transfected with small interfering RNA (siRNA) targeted to xanthine dehydrogenase (XDH), which is the precursor of XO. IH-induced XO and Nox activation were absent in cells transfected with XDH siRNA as compared with cells treated with scrambled siRNA (Fig. 2A-B).

### Ca^2+^ dependent PKC activation by IH requires XO

**Fig 3:**
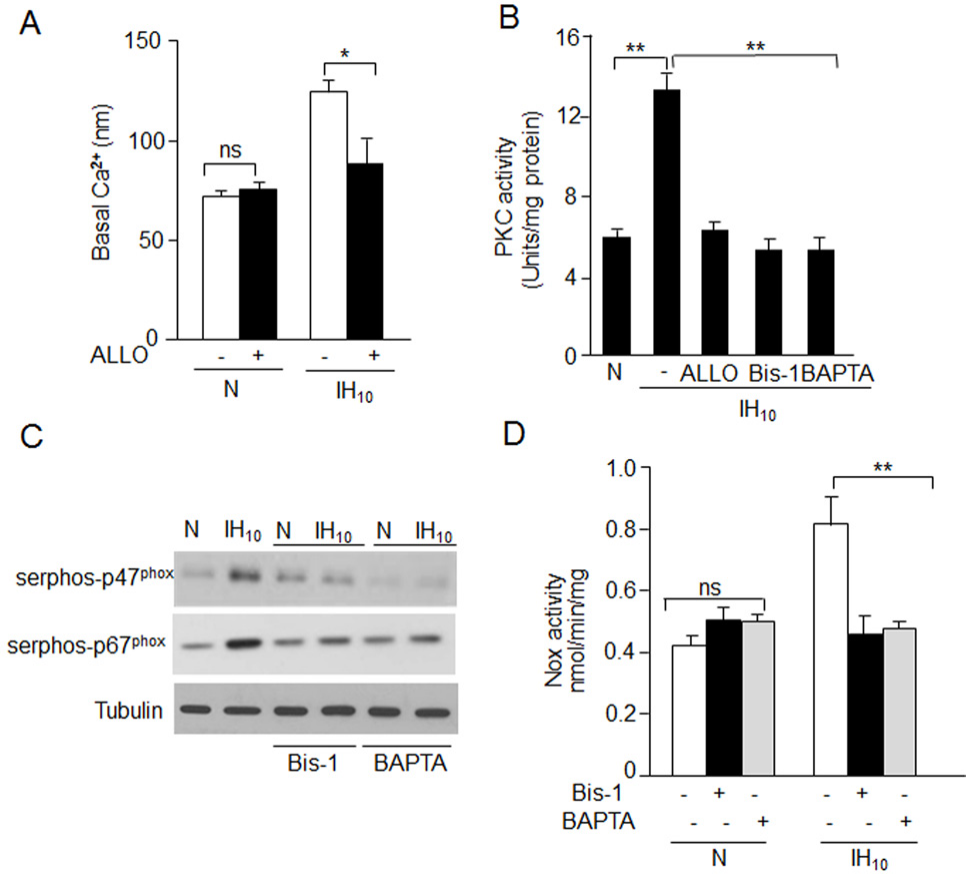
XO augments Nox stimulation via Ca^2+^ dependent PKC activation. A) [Ca^2+^]i levels were monitored in PC12 cells exposed to normoxia (N) or IH_10_ with or without ALLO treatment. B) Measurement of PKC activity in cells exposed to normoxia (N) or IH_10_ in presence of ALLO, bisindolymaleimide (Bis-1; inhibitor of multiple PKC isoforms, 10 μM) or BAPTA (intracellular calcium chelator, 10 μM). C) Representative immunoblot showing phosphorylation of p67^phox^ and p47^phox^ in presence of Bis-1 and BAPTA. D) Nox activity in cells exposed to normoxia (N) or IH_10_ in presence of BAPTA and Bis-1. Data are presented as mean ± S.E.M. from four experiments. ** p< 0.01; * p< 0.05; ns: not significant.

The mechanism(s) underlying XO-dependent Nox2 activation by IH were determined. Nox2 activation requires Ca^2+^-dependent PKC activation [14, 19]. Cells exposed to IH_10_ exhibited two-fold elevation in [Ca^2+^]_i_ levels and this effect was blocked by ALLO (Fig. 3A). We next determined whether IH_10_ elevated calcium levels activate PKC. In response to IH_10_, PKC activity increased 2.5 fold compared to control cells (P<0.01), and this effect was prevented by either ALLO, an inhibitor of XO; BAPTA-AM (10μM), a calcium chelator; or bisindolymaleimide (Bis-1; 10 μM), a pan PKC isoform inhibitor (Fig. 3B).

### XO mediates IH-evoked phosphorylation and translocation of p47^phox^ and p67^phox^

Nox2 activation requires PKC-dependent phosphorylation and subsequent translocation of the cytosolic p47^phox^ and p67^phox^ subunits [9] to the membrane. As shown in Fig. 3C, IH-exposed cells exhibited increased levels of phosphorylated p47^phox^ and p67^phox^ levels. Treating cells with either BAPTA-AM or Bis-1 prevented IH-induced phosphorylation of cytosolic subunits (Fig. 3C) as well as Nox activation (Fig. 3D).

**Fig 4:**
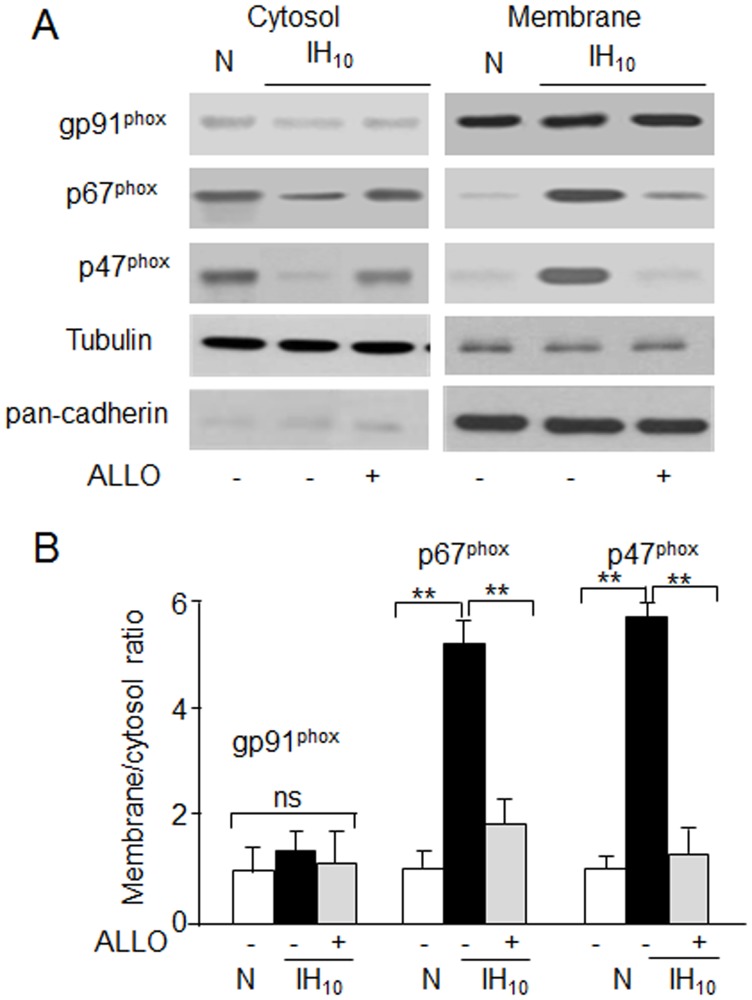
XO mediates IH-evoked translocation of p47^phox^ and p67^phox^. A) Representative immunoblot showing the distribution of gp91^phox^, p67^phox^ and p47^phox^ proteins in cytosolic and membrane fractions from cells exposed to normoxia (N) or IH_10_ with or without ALLO. B) Densitometric analysis of three immunoblots presented as mean ± S.E.M. Membrane to cytosol ratio was calculated for each protein after normalizing to tubulin and pan-cadherin respectively. Data is presented as mean ± S.E.M from three independent experiments. ** p<0.01; ns: not significant.

To demonstrate the translocation of the cytosolic subunits to the plasma membrane, the distribution of p67^phox^ and p47^phox^ proteins was analyzed in cytosolic and plasma membrane fractions of PC12 cells exposed to normoxia or IH along with gp91^phox^. The cytosol and membrane fractions were confirmed with the relative abundance of α-tubulin and pan-cadherin proteins, respectively (Fig. 4A). In normoxic cells, gp91^phox^ was localized predominantly in the membrane fraction, whereas p67^phox^ and p47^phox^ were abundant in the cytosolic fraction (Fig. 4A). While IH had no effect on gp91^phox^, it increased p67^phox^ and p47^phox^ levels in the membrane fraction with concomitant reduction in the cytosolic fraction (Fig. 4A-B). Pre-treatment with ALLO prevented the effects of IH on the distribution of p67^phox^ and p47^phox^ proteins to the membrane (Fig. 4A-B).

### Nox2 complex formation by IH requires XO

**Fig 5:**
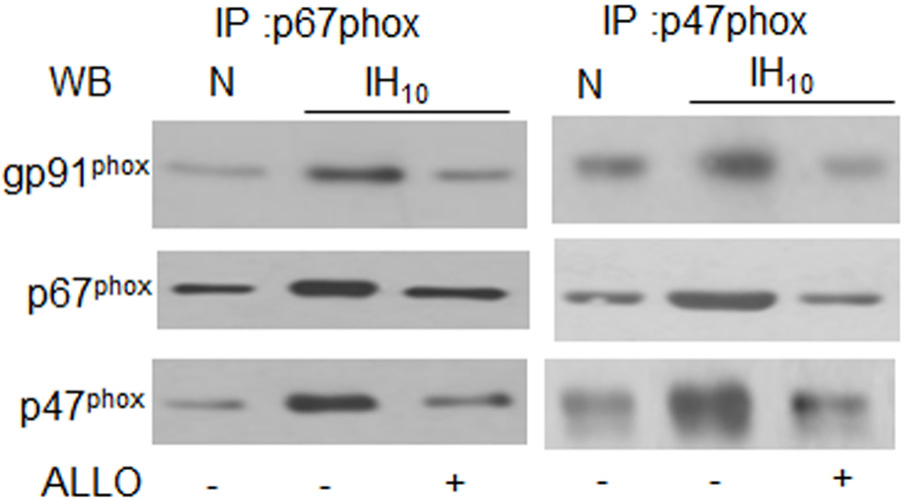
Nox2 complex formation by IH requires XO. Nox2 complex immunoprecipitated from membrane fraction of cells exposed to normoxia (N) or IH_10_ with p67^phox^ or p47^phox^ antibody and analyzed for gp91^phox^, p67^phox^ and p47^phox^ proteins by immunoblot.

The effect of IH on Nox2 complex formation was determined. To this end, p67^phox^ and p47^phox^ were immunoprecipitated and immunoblot assays were performed with anti-gp91^phox^, p67^phox^ and p47^phox^ antibodies. As shown in Fig. 5, amount of gp91^phox^ co-immunoprecipitated with p67^phox^ and p47^phox^ increased in IH-exposed cells as compared to cells exposed to normoxia, indicating formation of the active Nox2 complex. Likewise, p67^phox^ was detected in p47^phox^ immunoprecipitates and vice versa demonstrating the association of all three subunits. Treatment with ALLO completely abolished Nox2 complex formation in cells subjected to IH (Fig. 5).

### XO contributes to HIF-1α upregulation by IH

**Fig 6:**
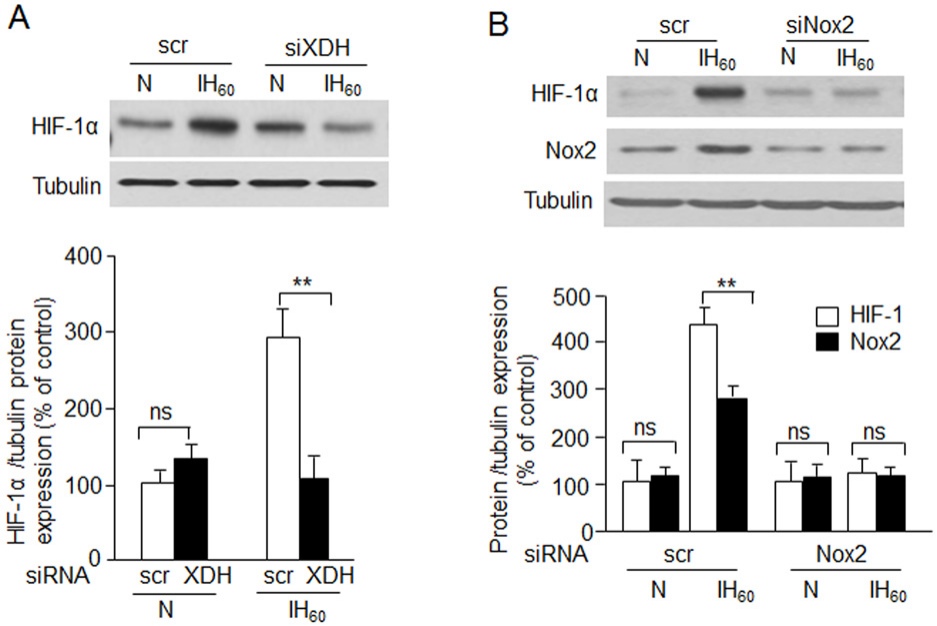
XO contributes to HIF-1α upregulation by IH. A) Top panel: Representative immunoblot showing the effects of XO silencing on HIF-1α protein expression in cells exposed to IH_60_. Bottom panel: densitometric analysis of three immunoblots presented as mean ± S.E.M. B) Representative immunoblot showing HIF-1α and Nox2 expression in cells transfected with scrambled (scr) siRNA or Nox2 siRNA and exposed to normoxia (N) or IH_60_ with or without ALLO treatment. Data are presented as mean ± S.E.M. from four experiments. ** p < 0.01; ns: not significant.

Our previous studies showed that Nox2 is required for HIF-1α activation by IH [4]. The results described above demonstrate that XO mediates IH-evoked Nox activation. Therefore, we tested the effects of silencing XO activation on HIF-1α activation by IH_._ Since significant HIF-1α activation was seen with IH_60_ but not IH_10_, HIF-1α protein expression was analyzed in IH_60_ exposed cells transfected with siRNAs targeted to either XDH or gp91^phox^. IH-evoked HIF-1α activation was completely prevented in cells treated with XDH or gp91^phox^ siRNA (Fig. 6A-B).

## Discussion

In the present study, we delineated the signaling mechanisms underlying Nox2 activation by IH. The following findings demonstrate that XO activation is the initial trigger for Nox2 activation by IH: first, IH-induced XO activation precedes Nox2; second, pharmacological blockade or genetic silencing of XO abolished Nox2 activation by IH; and third, XO activation increased Nox activity under normoxia, mimicking the effects of IH.

Our results provide insight into how XO stimulates Nox activity. In both vascular endothelial and smooth muscle cells, Nox2 activation requires translocation of the cytosolic subunits to the membrane via [Ca^2+^]_i_-activated PKC [19, 20]. Consistent with this possibility, we found that IH increases Ca^2+^-dependent PKC activation in PC12 cells and this effect was prevented by blockade of XO activity. More importantly, our results further demonstrated robust PKC–dependent phosphorylation of p47^phox^ and p67^phox^, and translocation to the plasma membrane in IH-exposed cells. Co-immunoprecipitation experiments provided direct evidence for Nox2 complex formation during IH, which was dependent on XO activation. PKC α, β, δ, and ζ are all known to phosphorylate p47^phox^ (14), whereas only PKC ζ is known to target p67^phox^ [21]. Further studies are necessary to delineate the relative contribution of PKC isoforms to the phosphorylation of Nox2 cytosolic subunits during IH. Interestingly, IH_10_ not only increased phosphorylation of p67^*phox*^ and p47^phox^ but also increased their relative expression. One possible explanation could be that complexing with gp91^phox^ at the membrane or changing the phosphorylation states of the individual components may increase their stability as previously proposed [20]. Pharmacological treatments that inhibited the subunits from translocating to the membrane also prevented their increased expression, supporting the proposed hypothesis. Further investigation is required to confirm this model. An important finding of this study is that IH-induced XO activity also contributes to increased levels of HIF-1α. We previously reported that IH-evoked HIF-2α degradation is mediated by Ca^2+^-dependent calpain activation via XO-generated ROS [13]. Current results demonstrate that the same Ca^2+^ signaling triggered by XO also mediates IH-evoked HIF-1α activation by stimulating Nox2. XO activation by IH is mediated by proteolytic conversion of XDH to XO which is blocked by inhibiting proteolysis with a trypsin inhibitor [13]. These findings are reminiscent of an early study showing that XDH can be converted to XO within minutes in response to ischemia [22] and suggests that similar rapid proteolytic conversion of XDH to XO during the initial cycles of the IH paradigm may play a regulatory role in triggering Nox2 activation. Previous studies with ischemia re-perfusion models showed that XO gets activated during the re-oxygenation phase rather than during ischemia. Similar to ischemia-reperfusion, IH is associated with alternating cycles of hypoxia and re-oxygenation. Since such re-oxygenation periods are absent during sustained hypoxia, we believe XO-NOX-HIF-α pathway is selective to IH.

**Fig 7:**
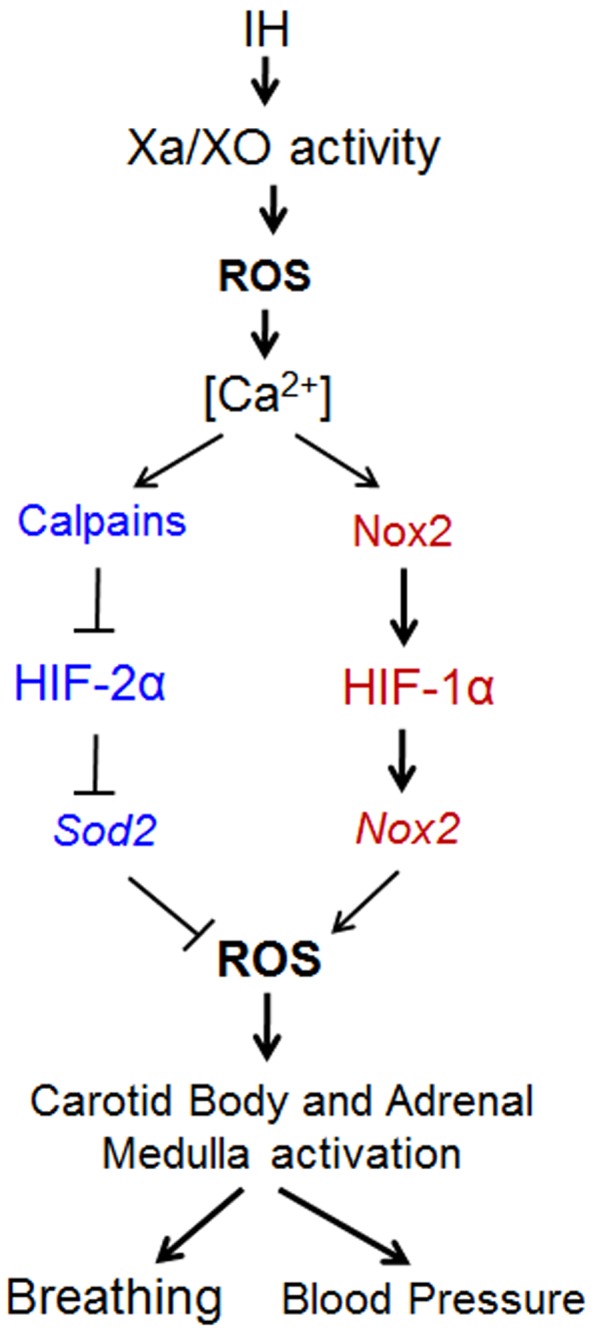
Schematic representation of feed-forward ROS-induced ROS mechanism by XO during IH. Exposing PC12 cells to IH_10_ activates XO, which triggers a series of events that lead to more persistent ROS production by increasing NOX and calpain activity and causes an imbalance in the HIFs that favors transactivation of pro-oxidant enzymes over anti-oxidant ones. Increased ROS levels lead to breathing abnormalities and hypertension.

It was proposed that increased generation of ROS mediates the pathological effects of IH [23]. Indeed, ROS levels were elevated in the carotid body [24], adrenal medulla [5, 25, 26] and central nervous system [27, 28] of rats and mice exposed to chronic IH. Remarkably, the effects of IH on catecholamine secretion, blood pressure elevation, long-term facilitation of respiratory motor activity, and sensory long-term facilitation in the carotid body can all be blocked by treating rodents with a ROS scavenger [29–33]. Obstructive sleep apnea patients exhibit elevated ROS levels [34–36] with impaired vasodilation, and antioxidant treatment restores the vascular responses [37]. These studies suggest that ROS play a critical role in mediating the cardiovascular pathology associated with IH. We previously reported that IH increases HIF-1α and decreases HIF-2α protein levels [25]. HIF-1 mediates transcriptional activation of Nox2, a pro-oxidant, whereas HIF-2 is a potent activator of genes encoding anti-oxidants including superoxide dismutase-2 [38]. Taken together, results from the current and previous studies, it is likely that XO-dependent ROS mediates imbalance between HIF-1 and HIF-2 via Nox2 and calpain activation, which leads to further long-lasting oxidative stress during IH that results in autonomic morbidities as summarized in Fig. 7.
