# Expression of Concern: HIF-1α Activation by Intermittent Hypoxia Requires NADPH Oxidase Stimulation by Xanthine Oxidase

**DOI:** 10.1371/journal.pone.0299122

**Published:** 2024-02-14

**Authors:** The PLOS ONE Editors

Following the publication of this article [1], concerns were raised regarding Figs 4 and 5. Specifically, there appears to be vertical discontinuities in the following locations:

- Fig 4A, Cytosol p47^phox^ panel, between lanes 1/2 and 2/3.
- Fig 4A, Membrane Tubulin panel, between lanes 1/2.
- Fig 5, IP: p47^phox^ panel, between lanes 1/2 and 2/3.

The corresponding author stated that primary data underlying the published results remain available except for the original images underlying Fig 4 and the IP: p47^phox^ panel in Fig 5. In the absence of the original data for these figures, they could not comment on whether the panels listed above were spliced.

The corresponding author provided uncropped images described as replicate data for Fig 4A and individual-level quantitative data underlying the graph in Fig 4B (included with this notice as S1 and S2 Files).

The corresponding author also provided uncropped images described as the original data for Fig 5 IP: p67^phox^ panels (S3 File) and original uncropped images corresponding to the published IP: p47^phox^, gp91^phox^, and p67^phox^ panels in Fig 5 (S4 File). For the IP: p67^phox^ panels, it was not possible to be certain that these correspond to the published panel due to poor image quality.

For Figs 4 and 5, the replicate or original data provided lend support for the reported results. However, without the original image data underlying the published figures, the concerns cannot be fully resolved.

The *PLOS ONE* Editors issue this Expression of Concern to notify readers of the above concerns which may impact the reliability of the conclusions drawn from these figures, and to relay the supporting data provided by the corresponding author.

## Supporting information

S1 File Replicate results for Fig 4A. (ZIP)

S2 File Quantitative data underlying Fig 4B. (XLSX)

S3 File Images described as the original data for Fig 5 IP: p67^phox^ panels. gp91^phox^ is also known as Nox2.
(ZIP)

S4 File Original images underlying Fig 5 IP: p47^phox^, except the p47^phox^ panel. gp91^phox^ is also known as Nox2.
(ZIP)
