# Supplementary figures and images for: Expression of Concern: HIF-1α Activation by Intermittent Hypoxia Requires NADPH Oxidase Stimulation by Xanthine Oxidase
Source: PLoS One. 2024 Feb 14;19(2):e0299122. doi: 10.1371/journal.pone.0299122 (PMC10866470; doi:10.1371/journal.pone.0299122)

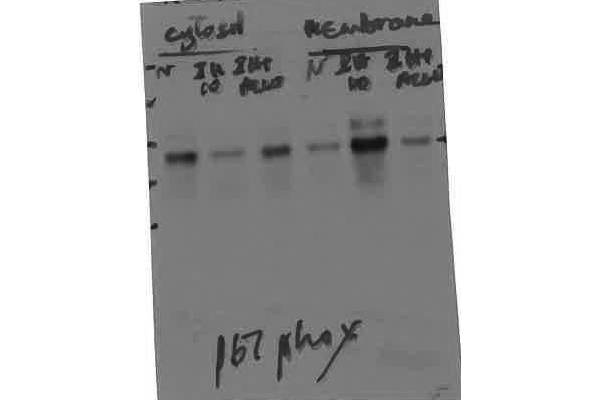

Supplement: S1 File — (ZIP) [file pone.0299122.s001.zip › fig 4A p67phox cyto and mem.tif]

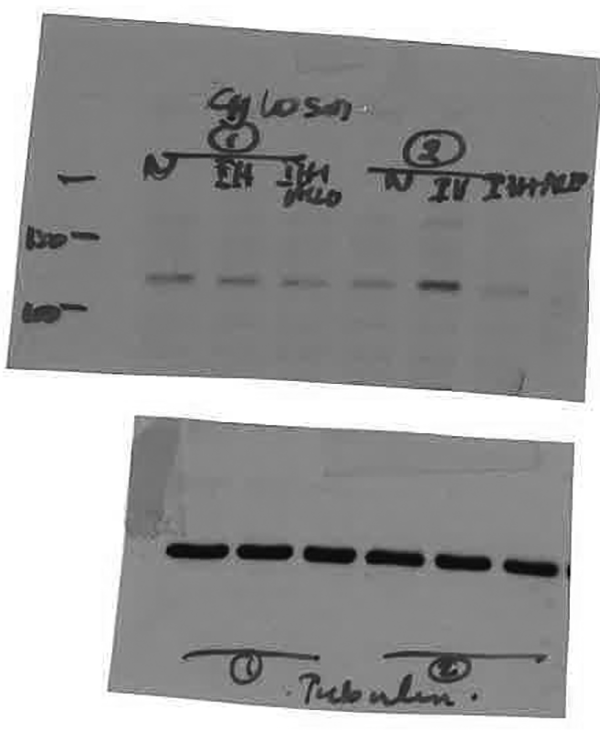

Supplement: S1 File — (ZIP) [file pone.0299122.s001.zip › Fig 4A tubulin and cadherin in cytosol.tif]

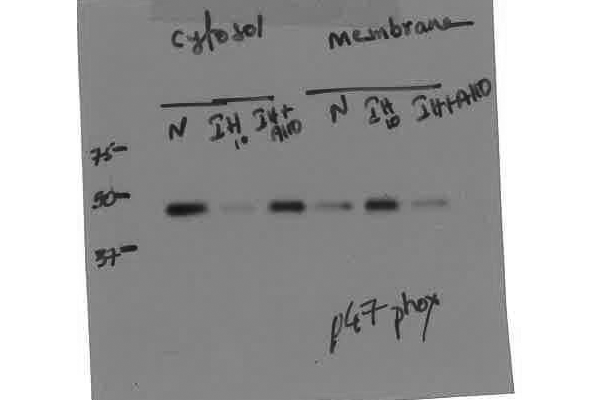

Supplement: S1 File — (ZIP) [file pone.0299122.s001.zip › fig 4Ap47phox cyto and membrane.tif]

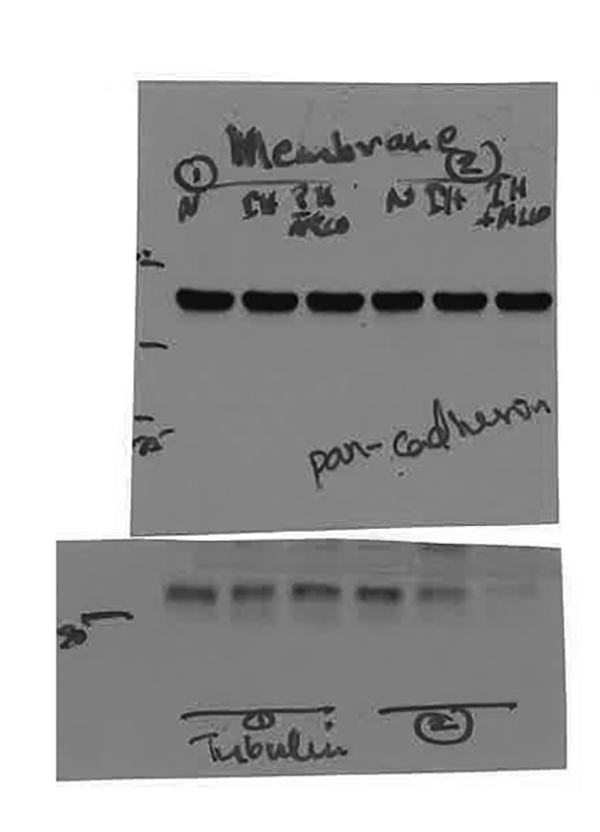

Supplement: S1 File — (ZIP) [file pone.0299122.s001.zip › Fig 4Atubulin and cadherin in membrane.tif]

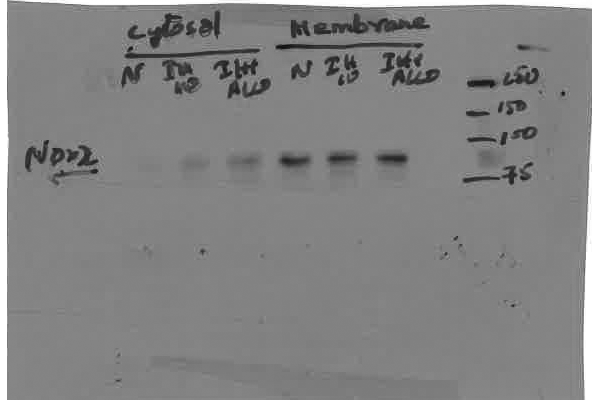

Supplement: S1 File — (ZIP) [file pone.0299122.s001.zip › Fig4A gp91phox cytoand mem.tif]

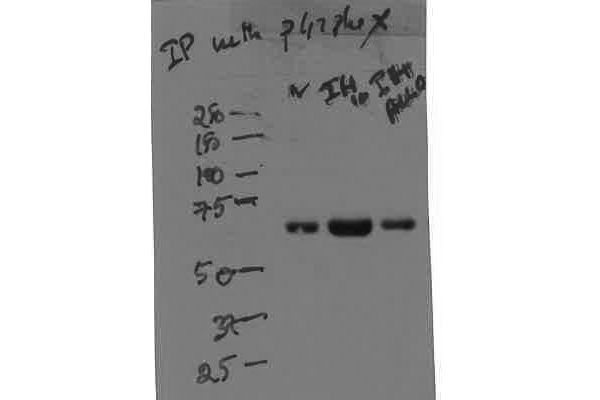

Supplement: S3 File — gp91phox is also known as Nox2. (ZIP) [file pone.0299122.s003.zip › S3_fig5 ipwith p47phox raw images/2Fig5 ipwith p47phox p67phox.tif]

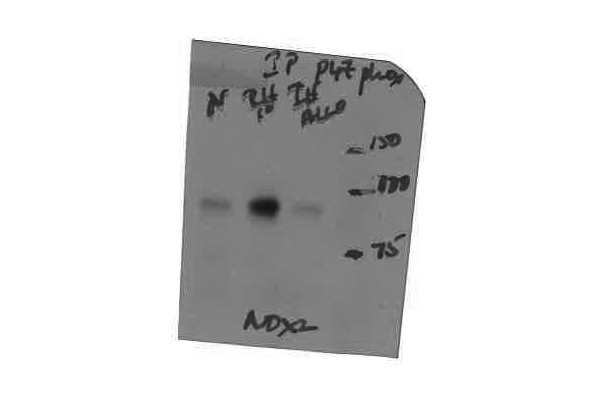

Supplement: S3 File — gp91phox is also known as Nox2. (ZIP) [file pone.0299122.s003.zip › S3_fig5 ipwith p47phox raw images/2fig5 ipwith p47phoxnox2.tif]

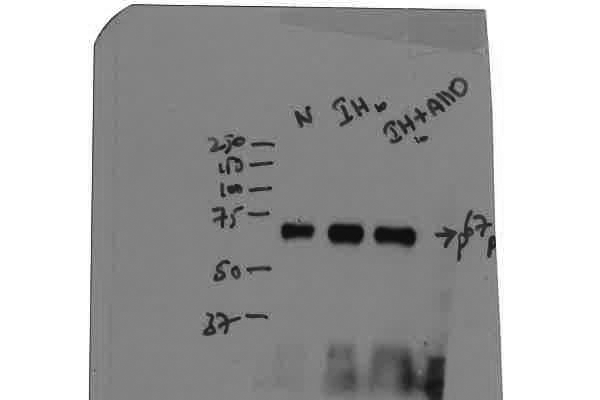

Supplement: S4 File — gp91phox is also known as Nox2. (ZIP) [file pone.0299122.s004.zip › S4_Fig5ipwith p67phox raw images/Fig 5ip with p67phox p67phox.tif]

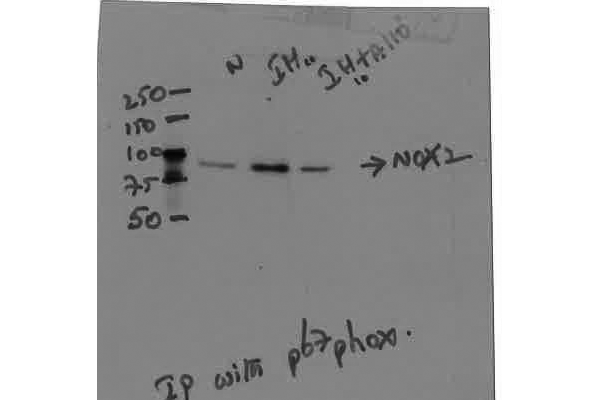

Supplement: S4 File — gp91phox is also known as Nox2. (ZIP) [file pone.0299122.s004.zip › S4_Fig5ipwith p67phox raw images/Fig5ipwith p67phox nox2.tif]

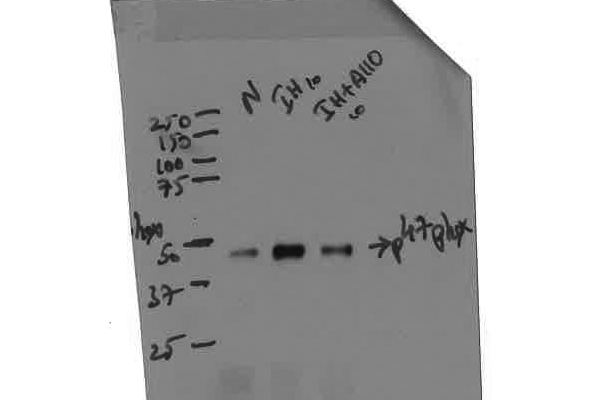

Supplement: S4 File — gp91phox is also known as Nox2. (ZIP) [file pone.0299122.s004.zip › S4_Fig5ipwith p67phox raw images/Fig5ipwith p67phox p47phox.tif]
